# A Reply to Dr. Battey and a Disclaimer

**Published:** 1887-03

**Authors:** Lawson Tait

**Affiliations:** Birmingham, England


### Correspondence.

### A REPLY TO DR. BATTEY AND A DISCLAIMER.

BY LAWSON TAIT, BIRMINGHAM, ENGLAND.

SIRS—The discussion on the subject of the operation which, by preference, I call "removal of the uterine appendages," seems to be endless, but I propose to carry it on as patiently as I can so long as I have strength to do it, and so long as I am misrepresented, as I have again been by Dr. George Battey in the current number of your journal, and misrepresented in a way that is wholly inexcusable.

Speaking of "Battey's operation," that is, normal ovariotomy, or the removal of ovaries which are not diseased, performed, in Dr. Battey's own original defining words, "merely to bring about the menopause," Dr. Battey says that "in Great Britain the operation has been very much done within the last ten years, I might say within the last eight years, by Dr. Tait, Dr. Savage and Dr. Malins, the three principal operators." I leave my two colleagues to speak for themselves, but as regards my own practice I beg to give Dr. Battey's statement the most emphatic contradiction. I have done the operation he advocates only some ten or eleven times, and this he must know if he reads the literature of gynæcology at all. In the last edition of my book on diseases of the ovaries, published in 1883 by Wood, of New York, and too extensively read in America to have escaped Dr. Battey's notice, I say (p. 326): "For an operation performed merely to 'bring about the menopause,' to quote Dr. Battey's own words, there must be but a limited field, and my own experience of it would be vague and indefinite, and my own conclusion would be doubtful." Since then I have placed on record my conclusions about it as so disappointing that I shall have no more to do with it.

It is alleged that this operation is being performed in England improperly, and legal proceedings have been taken in consequence, and therefore you will understand how necessary it is for me to assert the inaccuracy of Dr. Battey's statement, and to assure your readers that every one here objects to his operation. It has not a friend in the island. The two men he quotes as its friends, Spencer Wells and Matthews Duncan, are the bitterest opponents it has.

Further on Dr. Battey says: "A few years ago an eminent surgeon put before the profession the ingenious theory that the ovaries were organs of comparative little importance in the female economy; that the great central organ which determined the physiological process of menstruation was not the ovary but the fallopian tube." I have a suspicion that this sentence is directed at me. If I am wrong, I apologize in anticipation to Dr. Battey, but if it is, he ought to apologize to me for the misapprehension of my views, and writing on the subject is so outrageous as to be almost incredible. What I have said is that the ovaries have nothing to do with menstruation as a cause, and that I say still. But to attribute that to me as an original remark is to display an ignorance of the literature of the subject so stupendous that I can hardly believe my eyes as I read it. Ritchie, of Glasgow, proved it before I was born. Reeves Jackson, of Chicago, confirmed the proof before I entered the profession, and every real investigator since has established the conclusion. My own surgical work, as I have often said, goes all in the same direction. Further, I found that removal of the tubes without the ovaries often arrested menstruation completely; more than this I never said; therefore I concluded that they had more to do with menstruation than had ever before been suspected. Dr Battey further says: "I think I may safely say that the tubular theory of menstruation has fairly fallen to the ground. If anybody defends it now, I do not know the fact." Dr. Battey ought to have known that nobody ever tried to raise the dead horse he has been so industriously thrashing. All that intelligent workers in pelvic pathology and physiology desire, so far, is that people like Dr. Battey should read the elementary facts of the subject in such simple and well-known works as Ritchie's "Ovarian Physiology and Pathology," or Reeves Jackson's masterly paper, "Is the Ovulation Theory of Menstruation Tenable?" It would be too much to expect him to master the treatise of DeSwietz and Melassery.

The fact is that Dr. Battey's simple-minded physiology has regarded woman's complicated structure as a mere egg-laying machine. Stop the egg-laying if there is anything wrong, shouts Dr. Battey, and all will be right. This would have been a royal road to success if simplicity, either in men or measures, ruled the world's ways. But unfortunately it is not so, and the result has been disaster, and, in this country, a fearful and unnecessary row. "Battey's operation," "spaying," "normal ovariotomy" has rushed from mouth to mouth and incredible, disgraceful confusion has ensued, fostered largely by inducements of a meaner kind.

Speaking again of the eminent surgeon, whom he so completely misrepresents in matters physiological and pathological, Dr. Battey says: "He boldly claimed that if the fallopian tubes (Dr. Battey evidently does not know that these structures are named after Gabriel Fallope, and that therefore he ought to say if the Fallopian tubes) were removed the presence of the ovaries was a matter of indifference; that the change of life would attend upon the removal of the tubes simply, and that the curative results of the operation would be fully secured, leaving the ovaries behind. Unfortunately for this theory, it does not stand the test of experience." Here again, supposing always that Dr. Battey is alluding to me, he absolutely misrepresents the whole question, and simply because his mind is over-burdened with the idea that to do a patient any good, when she has something wrong in her pelvis, it is necessary to bring about the change of life—a view I do not share in the least. As a matter of fact, Dr. Battey and I have begun from the opposite corners of the sheet and we must always be face to face and at loggerheads. He began on the 17th of August, 1872, to work woman's welfare by removing healthy appendages in order to benefit her in a round-about way by bringing on his beloved change of life. On the absolute contrary, I began on February 11th, 1872, to remove uterine appendages for chronic inflammatory disease (a small ovarian abscess), thus breaking into the tradition of Spencer Wells that nothing was to be touched except big tumors.

I cared nothing about the change of life. I care nothing about it now. It generally comes when the appendages of both sides are diseased and removed, but many cases are cured in which it does not come, and very many are quite cured long before it does come.

Therefore the removal of the ovaries when the tubes require removal, either from inflammatory disease or on account of a uterine myoma, never troubles me. If they come away easily with the tube they are removed, and if not they stay. When Dr. Battey says that this is a theory, he is talking nonsense. When he says it is not a fact, he is doing something much more foolish.

I am sorry to have had to occupy so much of your space in correcting erroneous impressions concerning what may appear to be purely personal matters. I should not have troubled about them if their concern had been purely personal; but as I have had to take much trouble to correct misrepresentations about "Tait's operation," I must of necessity take quite as much to correct the statement that I perform or even approve of "Battey's operation."

It is far better and wiser not to use such meaningless and therefore such confusing phrases. I say there is no such thing as "Tait's operation," and—on this side of the Atlantic—there is many a poor wretch who wishes there had never been heard of such a procedure as "Battey's."

I am, etc., Lawson Tait.

*Birmingham, January 21, 1887.*

